# Mothers of children with food allergies report poorer perceived life status which may be explained by limited career choices

**DOI:** 10.1186/s13223-021-00515-8

**Published:** 2021-02-01

**Authors:** Tara Lynn Mary Frykas, Michael Golding, Elissa M. Abrams, Elinor Simons, Jennifer Lisa Penner Protudjer

**Affiliations:** 1 Department of Food and Human Nutritional Sciences, University of Manitoba, 501G-715 McDermot Ave, Winnipeg, MB R3E 3P4 Canada; 2 The Children’s Hospital Research Institute of Manitoba, Winnipeg, Canada; 3 Department of Pediatrics, University of Manitoba, Winnipeg, Canada; 4 Department of Pediatrics and Child Health, Section of Allergy and Clinical Immunology, University of Manitoba, Winnipeg, Canada; 5 George and Fay Yee Centre for Healthcare Innovation, Winnipeg, Canada; 6 Institute of Environmental Medicine, Karolinska Institutet, Stockholm, Sweden

**Keywords:** Children, Costs, Food allergy, Mothers, Perceived life status, Visual analogue scale

## Abstract

Pediatric food allergy is associated with direct, indirect and intangible costs. However, it remains unclear if intangible costs of pediatric food allergy influence parental career choices. Using data from 63 parents whose children had been diagnosed by a pediatric allergist with food allergy, we sought to (a) establish perceived life status of families with a food allergic child, and (b) to describe any career limitations viewed as attributable to food allergy. Compared to responding parents whose children had one to two food allergies, those with three or more food allergies had significantly poorer perceived life status (ß − 0.74; 95%CI − 1.41; − 0.07; p < 0.05). Overall, 14.3% of parents (all mothers) reported career limitations due to food allergy. Two of the 7 mothers (28.6%) who reported career limitations due to their child's food allergy fell below Statistics Canada cut-off for low-income, after tax dollars (LIM-AT). One of the three mothers who had changed jobs because of their child's food allergy was below the LIM-AT. No fathers reported food allergy-related career limitations. In conclusion, mothers of children with multiple food allergies reported worse perceived life status that may be partly explained by food allergy-related career limitations.

An estimated 7% of Canadian children live with food allergy [1]. In addition to a measurable financial burden [2], pediatric food allergy results in non-monetary intangible costs, such as opportunity and time losses [3, 4]. Intangible costs, which have no measurable monetary value, are associated with satisfaction and performance, and have psychosocial consequences (5). To date, it has not been studied whether the intangible costs, including perceived life status (self-reported score from 0 = worst, to 10 = best life possible) [6] of pediatric food allergy influence parental career choices. To this end, we performed a cross-sectional study to (a) establish perceived life status of families with a food allergic child, and (b) to describe any career limitations viewed as attributable to food allergy.

This study makes use of parent-reported data from the Food Allergy Economic Questionnaire (EcoQ) [4] collected between March 2019 and March 2020, during their children’s follow-up visits at a tertiary paediatric allergy clinic in Winnipeg, Canada. We included parents of children who had been diagnosed with food allergy by a pediatric allergist. We excluded parents whose children had other chronic diseases, including by not limited to diabetes and celiac disease, that impacted dietary choices and/or were not comfortable in answering an English-language questionnaire. To measure perceived life status, we used a visual analogue scale (VAS), which allowed participants to report their perceived life status on a scale of 0 (worst) to 10 (best), and that of their spouse (if applicable), the index child, and any of the child’s siblings. Parents were also queried about losses of career opportunities or restrictions specifically due to food allergy. Using parent-reported data on family size and income, we determined if families fell above or below the Low-Income Measure–After Tax dollars (LIM-AT) cut-off (Additional file 1: Table S3) [7].

Data were described using n/N, %, mean ± standard deviation (SD). To prevent right-skewing of data, we excluded one family with an annual income of  > $500,000. Linear regression was used to describe the impact of the number of food allergies (1–2 vs. 3 +) on perceived life status, with ß and 95th percent confidence intervals (95%CI) reported for unadjusted and adjusted models. Confounding variables included annual income, other atopic diseases for the child, and food allergy for the parent. Analyses were first considered in the entire study population. Then, we performed a sensitivity analysis in which we considered data reported by mothers only. Data were analysed with Stata 15.1 (College Station, TX). The University of Manitoba Health Research Ethics Board approved this study (HS22066 (H2018:319)).

Of the 63 families included, mothers most frequently completed the questionnaire (n = 55/63; 87.3%) (Additional file 1 Table S1). Amongst children (50.85% boys), the three most common allergies were peanuts/tree nuts (n = 50/63; 79.4%), eggs (n = 18/63; 28.6%) and fish (n = 14/63; 22.2%). Most children had developed food allergy in infancy (53/63; 84.1%) and had one to two food allergies (49/63; 77.8%). Nearly all (90.3%) had one or more other atopic condition(s) (asthma, eczema, dermatitis, rhinitis).

## Perceived life status and number of food allergies

Compared to responding parents whose children had one to two food allergies, those with three or more food allergies had significantly poorer perceived life status (ß − 0.74; 95% CI − 1.41; − 0.07; p < 0.05) (Table 1). No differences in perceived life status were seen for the spouse of the responding parent (p = 0.60), the child (p = 0.23), or siblings (p = 0.36). In a sensitivity analysis of mothers only, this difference persisted (ß − 0.88; 95%CI − 1.64; − 0.12; p < 0.05) (Additional file 1: Table S2).

**Table 1:** Perceived life status of families of children with 1–2 vs. 3 + food allergies (N = 63)

	Unadjusted		Adjusted^a^	
	ß	95% CI	ß	95% CI
Responding parent	− 0.74^†^	− 1.41; − 0.07	− 0.90^†^	− 1.59; 0.20
Spouse of responding parent	0.04	− 0.71; 0.78	− 0.10	− 0.90; 0.69
Child	− 0.80	− 1.65; 0.05	− 0.65	− 1.56; 0.25
Sibling	− 0.59	− 1.89; 0.72	− 0.24	− 1.56; 1.08

^a^Adjusted for annual income, other atopic diseases for child, food allergy for parent

^†^p < 0.05

## Mothers’ career limitations attributed to food allergy

Overall, 14.3% (8/55) of parents (all mothers) reported career limitations due to food allergy (Table 2), a finding which emphasizes the complex economic impact that pediatric food allergy has on caregivers (herein, exclusively mothers). Of the seven mothers who reported career limitations due to their child’s food allergy and for whom we had income data from which we calculated LIM-AT, 28.6% fell below the LIM-AT cut-off, [6], a proportion that was comparable to those who denied career limitations (25.5%). Annual household income was comparable between mothers whose career choices had (n = 7) vs. had not (n = 23) been restricted ($73,500 ± 30,777 vs. $84,021 ± 33,760; p = 0.50). Amongst mothers whose career choices had been limited, perceived life status was comparable between low-income (n = 2) and middle-income (n = 5) mothers (7.00 ± 0.00 vs. 7.60 ± 1.34; p = 0.18), 2/7 (28.5%) reported that their child had experienced anaphylaxis, 4/7 (57.1%) reported that their child had asthma, and 3/7 (42.9%) had at least one other household member with a food allergy. No fathers reported food allergy-related career limitations. Whereas lost labour market activity is difficult to quantify, an American study described that opportunity cost (defined as additional cost related to forgone labour activities) [8] to be $2399 USD annually per child, which surpassed all other food allergy-related costs combined [3]. Our study oversampled families below the LIM-AT, at 2.5 times the provincial average (33.3% vs. 13.4%) [6. The proportion of mothers who had career limitations due to their child’s food allergy may have been greater in a population of exclusively middle and high income families. Importantly, the provincial government subsidizes childcare fees to qualifying families, which would otherwise range between $20.80 to $45, depending on the age of the child and total daily hours spent at childcare [8].

**Table 2:** Mothers' career limitations^a^ due to food allergy (N = 49)

	n	%
Career choice restricted	7	14.3
Gave up job	2	4.1
Dismissed from job	0	0.0
Changed jobs	3	6.1

No fathers reported any career limitations due to food allergy

^a^Not mutually exclusive

In a review article published in 2017, Patel et al. noted that the burden of pediatric food allergy transcends all aspects of a family’s life [10]. Our findings reaffirm those reported by Patel et al. [8], and provide evidence that mothers of children with food allergy may make career choices specifically because of their child’s allergy. Few studies have examined parental career limitations in association with child diseases. In a small Lebanese study (n = 37), more than 80% of parents reported that their career has been affected by their child’s Type I diabetes [11], an estimate which is much higher than identified in our sample of families with food allergy. As such, future studies are warranted to explore if these findings persist in other populations, in order to identify policy needs that will directly benefit affected families. In Canada, unlike other conditions, including celiac disease, that necessitate medical dietary restrictions, there are presently no disability tax credits available for families managing food allergy [12]. Our study underscores the substantial psychosocial and economic burden that parents (predominantly mothers) bear as a result of their child’s food allergy. For a small proportion of mothers, this burden gives rise to removal from the work force. Whereas this choice was only seen in our study for higher income families, it behoves us to consider the additional burden borne by lower income families who may not have the option to stop working to care for their child. For these families, food allergy-related disability tax credits may be particularly beneficial.

In conclusion, the food allergic child and other family members had similar perceived life status regardless of the number of food allergies, yet mothers of children with multiple food allergies reported worse perceived life status that may be partly explained by food allergy-related career limitations.

## Supplementary Information

**Additional file 1: Table S1. **Participant and family demographics (N = 63). **Table S2.** Perceived life status of families of children with 1-2 vs. 3+ food allergies, amongst mothers as responding parents (N = 55). **Table S3.** Low-income thresholds, based on after tax dollars, for private households of Canada, 2015 (Reference 6).

## Data Availability

The datasets analysed in the current study are not publicly available due to the real potential of identifying individual allergists in regions with few practicing allergists.

## Abbreviations

EcoQ: Food Allergy Economic Questionnaire
LIM-AT: Low-Income Measure–After Tax dollars
SD: Standard deviation
VAS: Visual Analogue Scale
95%CI: 95th percent confidence interval
