# Supplementary material for: Mothers of children with food allergies report poorer perceived life status which may be explained by limited career choices
Source: Allergy Asthma Clin Immunol. 2021 Feb 1;17:12. doi: 10.1186/s13223-021-00515-8 (PMC7852075; doi:10.1186/s13223-021-00515-8)
Supplement: Supplementary file 1 — Additional file 1: Table S1. Participant and family demographics (N = 63). Table S2. Perceived life status of families of children with 1-2 vs. 3+ food allergies, amongst mothers as responding parents (N = 55). Table S3. Low-income thresholds, based on after tax dollars, for private households of Canada, 2015 (Reference 6). [file 13223_2021_515_MOESM1_ESM.docx]

Table S1. Participant and Family Demographics (N=63)

| **Children** | **n** | **%** |
| --- | --- | --- |
| Sex (N=61) |  |  |
| Boys | 31 | 50.8 |
| Girls | 30 | 49.2 |
| Number of food allergies |  |  |
| 1-2 | 49 | 77.8 |
| 3+ | 14 | 22.2 |
| Top 3 food allergies |  |  |
| Peanuts and tree nuts | 50 | 79.4 |
| Eggs | 18 | 28.6 |
| Fish | 14 | 22.2 |
| Previous food allergy symptoms |  |  |
| Itch, rash (N=62) | 60 | 96.8 |
| GI issues, runny eyes/nose (N=60) | 54 | 90.0 |
| Shortness of breath (N=52) | 25 | 48.1 |
| LOC, hypotensive (N=48) | 14 | 29.2 |
| Other atopic conditions* (N=62) |  |  |
| None | 6 | 9.7 |
| At least one | 56 | 90.3 |
| Current age (N=62) | 6.7 ± 4.8 | |
| Diagnosed by age 2 years | 53 | 84.1 |
| **Responding Parent** |  |  |
| Sex |  |  |
| Male | 8 | 12.7 |
| Female | 55 | 87.3 |
| Current age | 37.3 ± 6.5 | |
| Food allergies | 8 | 12.7 |
| Either parent has food allergy | 18 | 28.6 |
| Single parent | 3 | 4.8 |
| Average family size | 3.9 ± 0.9 | |
| Average annual income† | 75188 ± 35570 | |
| Low-income threshold‡ | 19 | 28.3 |
|  |  |  |
| *Asthma, eczema, dermatitis, and/or rhinitis |  |  |
| †Excludes annual family incomes (n=2) in excess of $1,000,000 | | |
| ‡Low Income Measure Threshold, based on after tax dollars (LIM-AT), per Statistics Canada (Reference 6) | | |

Table S2. Perceived life status of families of children with 1-2 vs. 3+ food allergies, amongst mothers as responding parents (N=55)

|  | **Unadjusted** | | **Adjusted*** | |
| --- | --- | --- | --- | --- |
|  | ß | 95% CI | ß | 95% CI |
| Mothers | -0.68 | 1.41; 0.04 | **-0.88†** | **-1.64; -0.12** |
| Spouse of mother | 0.17 | -0.64; 0.97 | 0.03 | -0.85; 0.90 |
| Child | **-1.00†** | **-1.94; -0.06** | -0.87 | -1.89; 0.14 |
| Sibling | -0.56 | -1.89; 0.77 | -0.25 | -1.60; 1.10 |
|  |  |  |  |  |
| *Adjusted for annual income, other atopic diseases for child, food allergy for parent | | | | |
| †p<0.05 |  |  |  |  |

Table S3. Low-income thresholds, based on after tax dollars, for private households of Canada, 2015 (Reference 6)

| Size of family† | After-tax income |
| --- | --- |
| 2 | 31,301 |
| 3 | 38,335 |
| 4 | 44,266 |
| 5 | 49,491 |
| 6 | 54,215 |
| 7+ | 58,558 |
|  | |
| †It is assumed that the smallest family is one parent + 1 child (i.e. 2 people). | |
